# Licochalcone A Attenuates Oxidative Stress and Inflammation to Promote Second-Degree Burn Healing Through Nrf2/HO-1 Activation in Mice

**DOI:** 10.3390/ijms27219450

**Published:** 2026-10-23

**Authors:** Menglong Xu, Hangzhen Zhou, Shuwei Li, Yongchao Li, Qinqin Wang, Haocheng Guan, Xiaoxia Luo

**Affiliations:** Department of Biochemistry and Molecular Biology, College of Life Science and Technology, The State Key Laboratory Incubation Base for Conservation and Utilization of Bio-Resource in the Tarim Basin of the Xinjiang Production and Construction Corps, Tarim University, Alar 843300, China; xumenglong1989@163.com (M.X.); xj_zhz1997@126.com (H.Z.); yfzx_406@126.com (S.L.); 18850569602@163.com (Y.L.); 15899089407@163.com (Q.W.)

**Keywords:** Licochalcone A, burn wound healing, oxidative stress, inflammation, Keap1/Nrf2/HO-1

## Abstract

Burn injury represents one of the most prevalent accidental traumas worldwide, contributing to substantial morbidity and mortality. Second-degree burns are frequently complicated by bacterial infection, excessive inflammation, and oxidative stress, which collectively impede wound healing. Licochalcone A (LCA), a bioactive flavonoid isolated from licorice, exhibits anti-inflammatory and antioxidant properties, yet its therapeutic role in burn wounds remains unexplored. Here, we evaluated the effects of a topical LCA-containing hydrogel on wound healing in a mouse dorsal second-degree scald model (100 °C, 5 s) and explored the associated molecular changes. In vivo, LCA-hydrogel treatment was associated with increased collagen deposition, reduced expression of interleukin-1β (IL-1β), interleukin-6 (IL-6), and tumor necrosis factor-α (TNF-α), reduced neutrophil and macrophage infiltration, and upregulated Ki67 and vascular endothelial growth factor C (VEGF-C). In hydrogen peroxide (H_2_O_2_)-stimulated fibroblasts, LCA attenuated reactive oxygen species (ROS) accumulation, improved cell viability and migration, preserved mitochondrial integrity, and restored antioxidant enzyme activities. Integrated transcriptomic and proteomic analyses identified 227 co-differentially expressed genes/proteins enriched in the reactive oxygen species pathway. Western blotting showed that the effects of LCA were associated with increased nuclear factor erythroid 2-related factor 2 (Nrf2) and heme oxygenase-1 (HO-1) expression and decreased Kelch-like ECH-associated protein (Keap1) expression. These exploratory findings suggest that the LCA-containing hydrogel is associated with accelerated second-degree burn healing, and that the antioxidant effects of LCA are associated with modulation of Keap1/Nrf2/HO-1 signaling, supporting further evaluation of its potential as a burn therapeutic candidate.

## 1. Introduction

Burn injury is one of the most common accidental traumas encountered in clinical practice and poses a serious threat to human health, with approximately 11 million cases and 180,000 deaths occurring worldwide each year [1,2]. According to the three-degree and four-category classification, second-degree (partial-thickness) burns extend into the dermis and are characterized by intense pain, exudation, and a risk of scarring [1]. Such wounds are frequently complicated by bacterial infection, excessive inflammation, and oxidative stress, which markedly delay healing; severe cases may progress to systemic inflammatory response syndrome, sepsis, and multiple organ failure, and survivors often suffer long-term burdens such as scar contracture and psychological disorders [2,3,4,5]. Although considerable progress has been made in burn care, current treatments remain unsatisfactory [6,7]. Silver sulfadiazine, the most widely used topical antimicrobial, is cytotoxic to keratinocytes and fibroblasts and can delay re-epithelialization [8,9], while surgical excision and skin grafting are limited by donor-site morbidity [10,11]. Hydrocolloid and modern advanced dressings, while improved in moisture management, are often expensive, require frequent replacement, and do not actively promote regenerative healing [12]. There is therefore a need for safer and more effective wound-healing agents, and natural products represent a promising source [13].

Licochalcone A (LCA; C_21_H_22_O_4_, molecular weight 338.4) is a chalcone-type flavonoid isolated from the roots of licorice (Glycyrrhiza uralensis Fisch. ex DC.) that possesses anti-inflammatory, antioxidant, and antitumor activities [14]. Notably, LCA has demonstrated in vitro antibacterial activity against Staphylococcus aureus, including methicillin-resistant strains (MRSA), one of the most common pathogens complicating burn wound infections [15,16]. LCA suppresses inflammatory responses by inhibiting the Mitogen-Activated Protein Kinase (MAPK) and Nuclear Factor-κB (NF-κB) pathways in macrophages and microglia [17,18] and inhibits the growth of various tumor cells, including glioma, osteosarcoma, and breast and gastric cancer cells, through multiple signaling pathways [14,19]. Moreover, LCA alleviates acetaminophen-induced hepatotoxicity [20], attenuates neuroinflammation and chronic neuropathic pain [21], and counteracts ultraviolet- and visible light-induced oxidative stress and photodamage [22,23]. These pharmacological properties correspond closely to the pathological features of burn wounds; however, whether LCA promotes burn wound healing and the underlying mechanisms remain unclear.

Accordingly, the present study investigated whether topically applied LCA promotes second-degree burn wound healing in a murine thermal burn model and in H_2_O_2_-challenged L929 and A9 fibroblasts, and explored the underlying mechanisms through histological, biochemical, transcriptomic, proteomic, and molecular analyses.

## 2. Results

### 2.1. LCA Promotes Collagen Deposition in Burn Wounds

**Figure 1:**
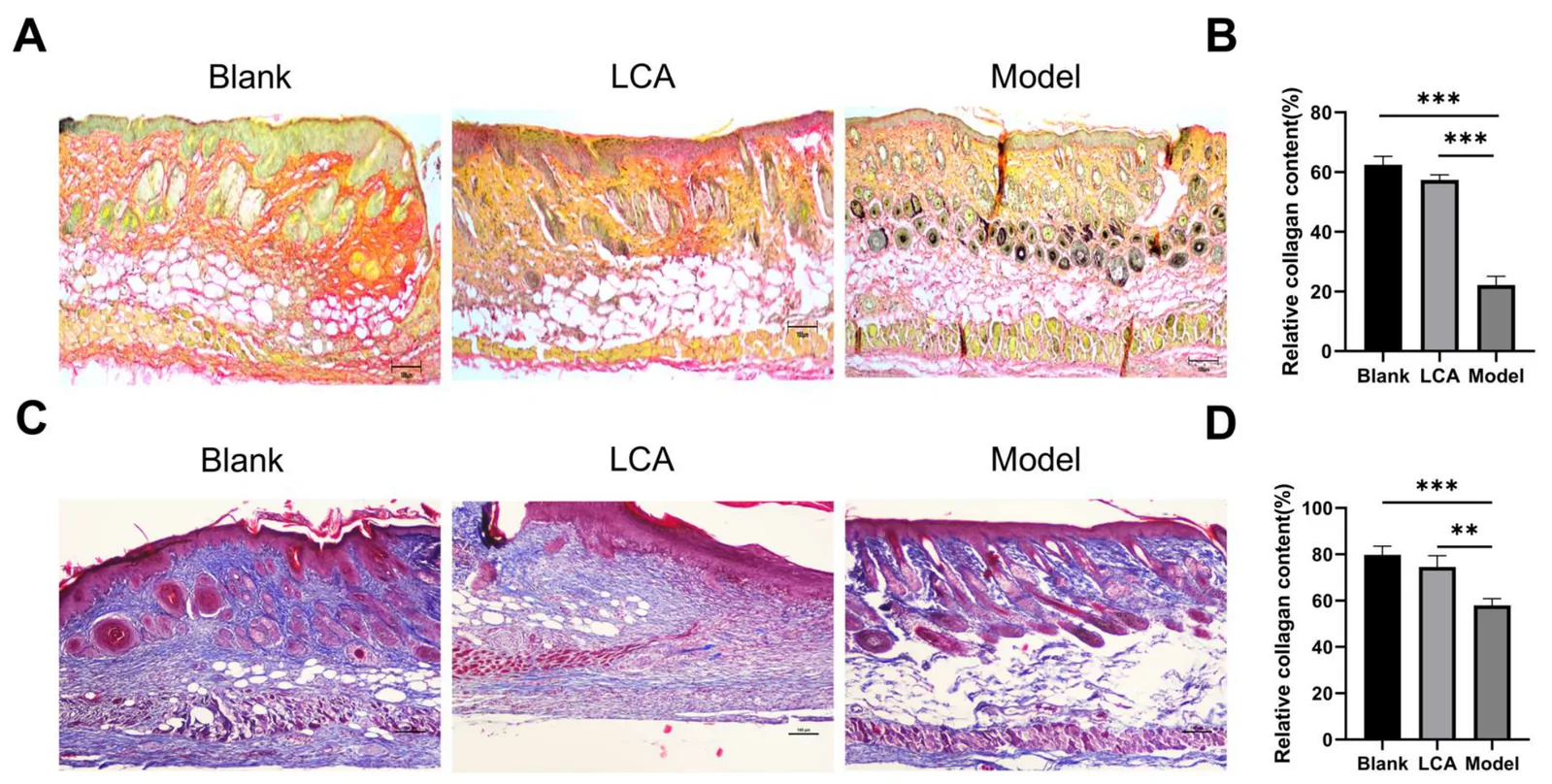
LCA promotes collagen deposition in burn wound skin. (**A**) Representative Sirius red staining. (**B**) Quantitative analysis of the relative collagen content from Sirius red staining. (**C**) Representative Masson’s trichrome staining. (**D**) Quantitative analysis of the relative collagen content from Masson’s trichrome staining. Scale bars = 100 μm. Data are expressed as mean ± SD (*n* = 3 mice per group; 3 sections per mouse, 5 fields per section); ** *p* < 0.01, *** *p* < 0.001.

Representative wound photographs and quantified wound area at days 3, 5, 7, and 9 post-burn (*n* = 3 mice per group per time point; different mice at each time point) showed that the wound area of the LCA group was smaller than that of the Model group at each time point (all *p* < 0.001, compared separately at each time point), and was similar to that of the MEBO group, with near-complete closure and hair regrowth by day 9 (Supplementary Figure S1A,B). These findings extend the day-9 histological and molecular results described below to the dynamic process of wound closure over time. Sirius red and Masson’s trichrome staining were performed on day-9 wound skin to assess collagen deposition at the tissue level. Sirius red staining showed relatively few yellow/orange-red type I collagen fibers in the Model group, whereas the LCA-treated wounds displayed a larger positively stained area (Figure 1B). Masson’s trichrome staining showed dispersed and fragmented collagen fibers in the Model group, whereas collagen fibers in the LCA group were denser and more compactly arranged (Figure 1D). Quantitative analysis confirmed that the relative collagen content was significantly higher in the LCA group than in the Model group (*p* < 0.001, Figure 1B; *p* < 0.01 and *p* < 0.001, Figure 1D), demonstrating that topical LCA promotes collagen synthesis and deposition during burn wound healing.

### 2.2. LCA Suppresses Inflammatory Cytokine Expression in Burn Wounds

**Figure 2:**
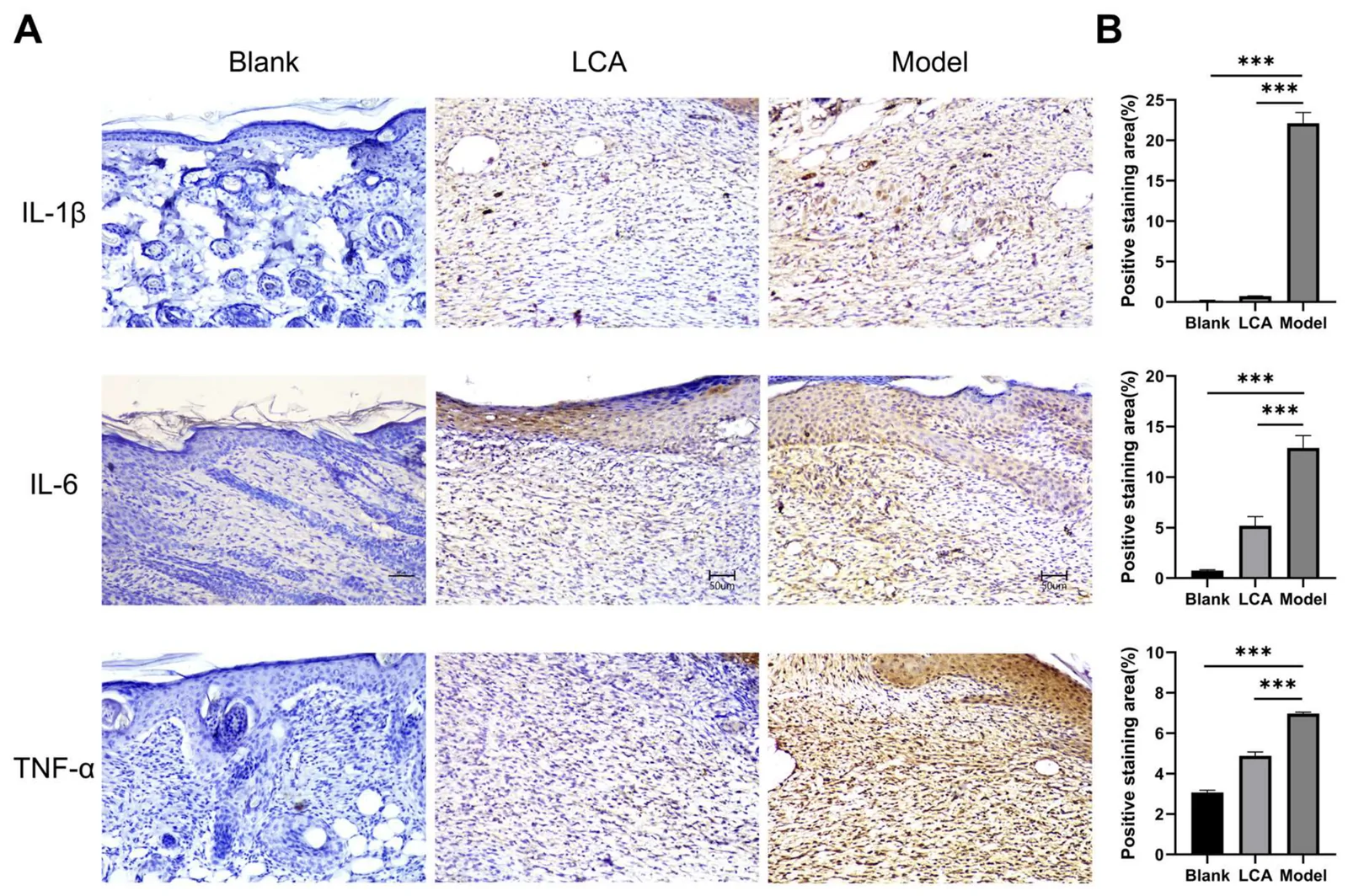
LCA suppresses pro-inflammatory cytokine expression in burn wound skin. (**A**) Representative immunohistochemical staining images of IL-1β, IL-6, and TNF-α in wound skin from the Blank, LCA, and Model groups. (**B**) Quantitative analysis of the positive staining area (%) of IL-1β (**top**), IL-6 (**middle**), and TNF-α (**bottom**). Scale bars = 50 μm. Data are expressed as mean ± SD (*n* = 3 mice per group; 3 sections per mouse, 5 fields per section); *** *p* < 0.001 (comparisons between the indicated groups).

Immunohistochemical staining of day-9 wound skin was performed to assess the distribution of the pro-inflammatory cytokines IL-1β, IL-6 and TNF-α. The positively stained areas of all three cytokines were markedly larger in the Model group than in the Blank group, reflecting robust local inflammation after burn injury. LCA treatment visibly reduced the distribution of IL-1β, IL-6 and TNF-α (Figure 2A), and quantitative analysis confirmed that the positive expression of each cytokine was significantly lower in the LCA group than in the Model group (all *p* < 0.001; Figure 2B). These results indicate that topical LCA suppresses the local inflammatory response in burn wound tissue.

### 2.3. LCA Reduces Inflammatory Cell Infiltration and Increases Ki67 and VEGF-C Expression

**Figure 3:**
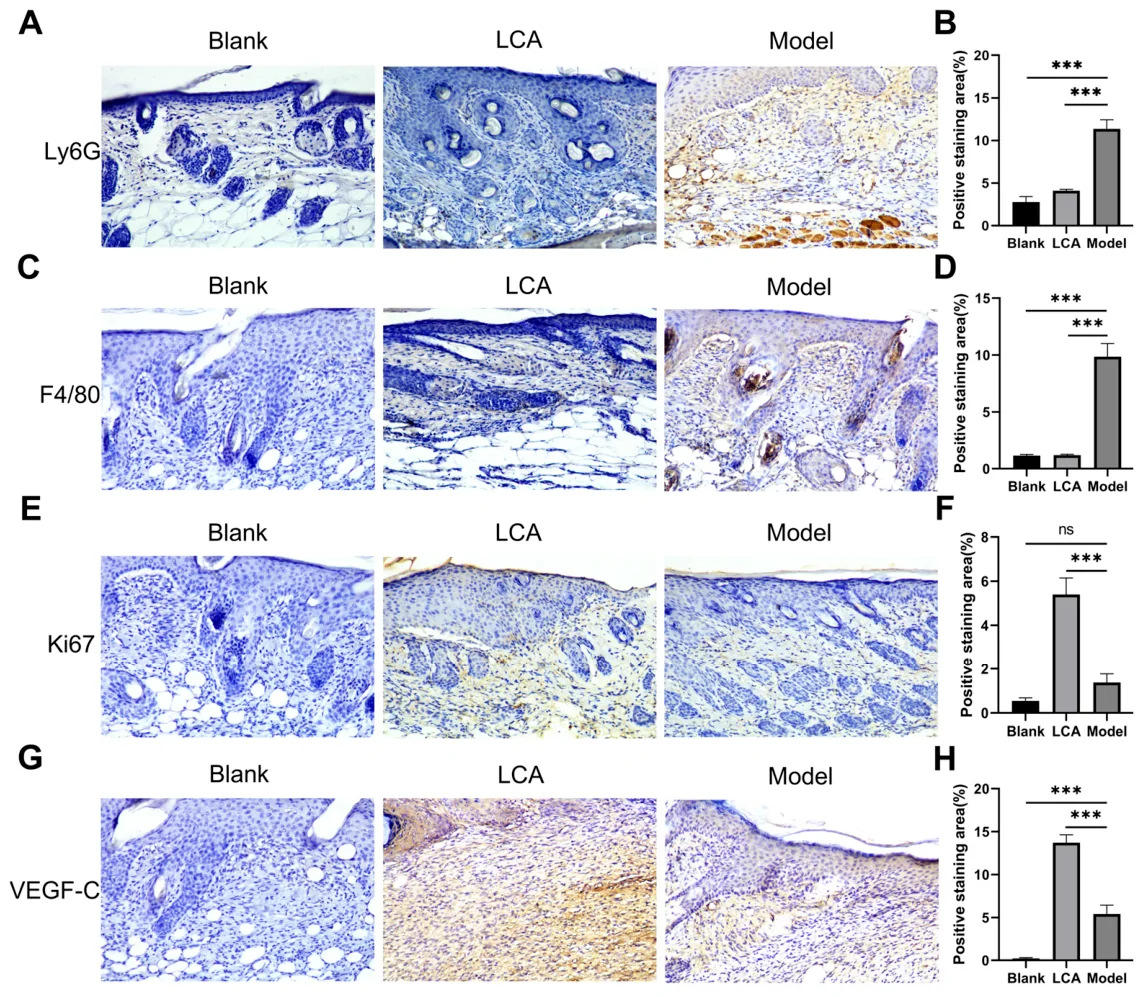
LCA reduces inflammatory cell infiltration and increases Ki67 and VEGF-C expression in burn wound skin. (**A**) Representative Ly6G immunohistochemical staining. (**B**) Quantitative analysis of Ly6G-positive staining area. (**C**) Representative F4/80 immunohistochemical staining. (**D**) Quantitative analysis of F4/80-positive staining area. (**E**) Representative Ki67 immunohistochemical staining. (**F**) Quantitative analysis of Ki-67-positive staining area. (**G**) Representative VEGF-C immunohistochemical staining. (**H**) Quantitative analysis of VEGF-C-positive staining area. Scale bars = 50 μm. Data are expressed as mean ± SD (*n* = 3 mice per group; 3 sections per mouse, 5 fields per section). ns, not significant, *** *p* < 0.001 vs. Model group.

The infiltration of neutrophils and macrophages was evaluated by immunohistochemical staining of Ly6G and F4/80, respectively. Massive infiltration of Ly6G^+^ neutrophils and F4/80^+^ macrophages was observed in the wounds of the Model group, whereas infiltration was substantially attenuated in the LCA group (Figure 3A,C); quantification showed that Ly6G and F4/80 positive expression was significantly lower in the LCA group than in the Model group (Figure 3B,D, both *p* < 0.001). In parallel, the positive expression of the proliferation marker Ki67 and the angiogenesis-related factor VEGF-C was markedly higher in the LCA group than in the Model group (both *p* < 0.001; Figure 3E–H). These findings suggest that LCA restrains excessive inflammatory cell infiltration while promoting proliferation and angiogenesis during burn wound healing.

### 2.4. Concentration Selection and Cytoprotective Effect of LCA In Vitro

**Figure 4:**
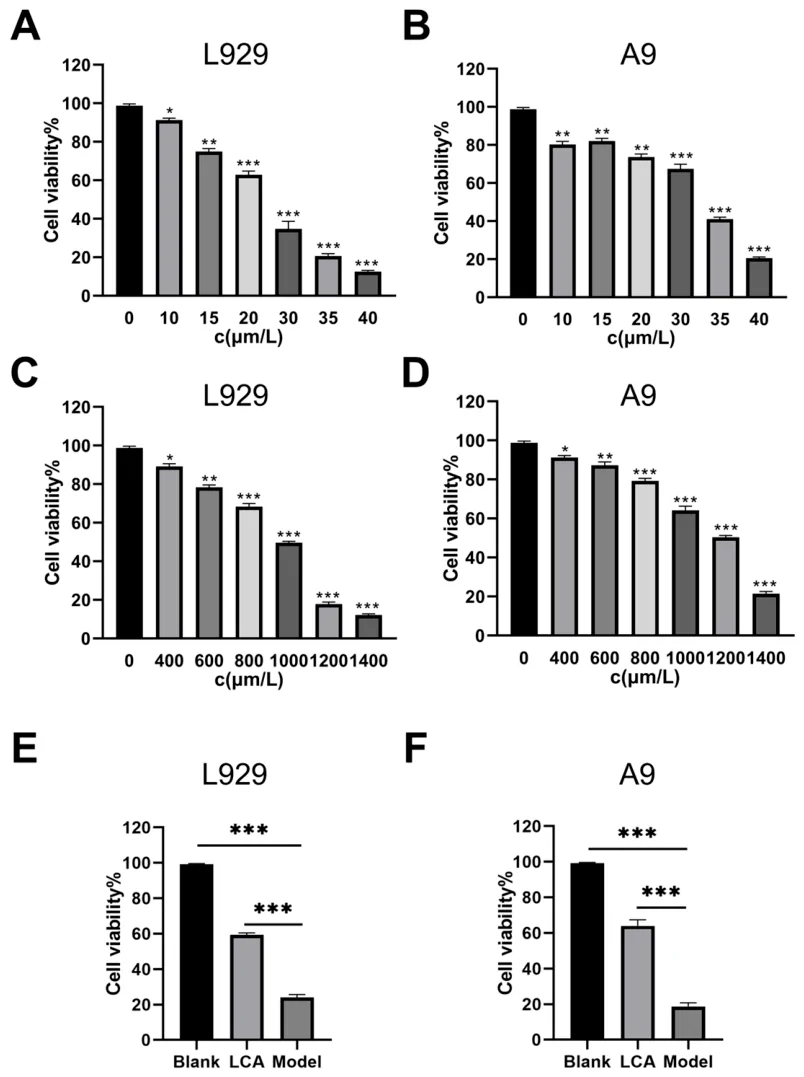
Concentration screening and cytoprotective effect of LCA in vitro. (**A**,**B**) Cell viability of L929 and A9 cells treated with gradient concentrations of LCA for 24 h determined by CCK-8 assay. (**C**,**D**) Cell viability of L929 and A9 cells exposed to increasing concentrations of H_2_O_2_ for 12 h. (**E**,**F**) LCA pretreatment ameliorated H_2_O_2_-induced cell damage in L929 and A9 fibroblasts. Data are expressed as mean ± SD. * *p* < 0.05, ** *p* < 0.01, *** *p* < 0.001.

To determine the appropriate working concentrations, L929 and A9 cells were exposed to increasing concentrations of LCA or H_2_O_2_, and cell viability was measured by the Cell Counting Kit-8 (CCK-8) assay. In L929 cells, viability was approximately 66% after 24 h of treatment with 20 μM LCA but dropped sharply at 30 μM; in A9 cells, viability fell below 50% at 30–35 μM (Figure 4A,B). Accordingly, 20 μM and 30 μM LCA were selected as the working concentrations for L929 and A9 cells, respectively. After 12 h of H_2_O_2_ stimulation, L929 cell viability was approximately 60% at 1000 μM and declined precipitously at 1200 μM, whereas A9 cell viability fell below 50% at 1200–1400 μM (Figure 4C,D); therefore, 1000 μM (L929) and 1200 μM (A9) H_2_O_2_ were used to establish the oxidative stress injury model. Pretreatment with LCA for 24 h before H_2_O_2_ exposure significantly increased the viability of both cell lines compared with the Model group (both *p* < 0.001; Figure 4E,F), demonstrating that LCA protects fibroblasts against H_2_O_2_-induced oxidative injury.

### 2.5. LCA Reduces Intracellular ROS Accumulation

**Figure 5:**
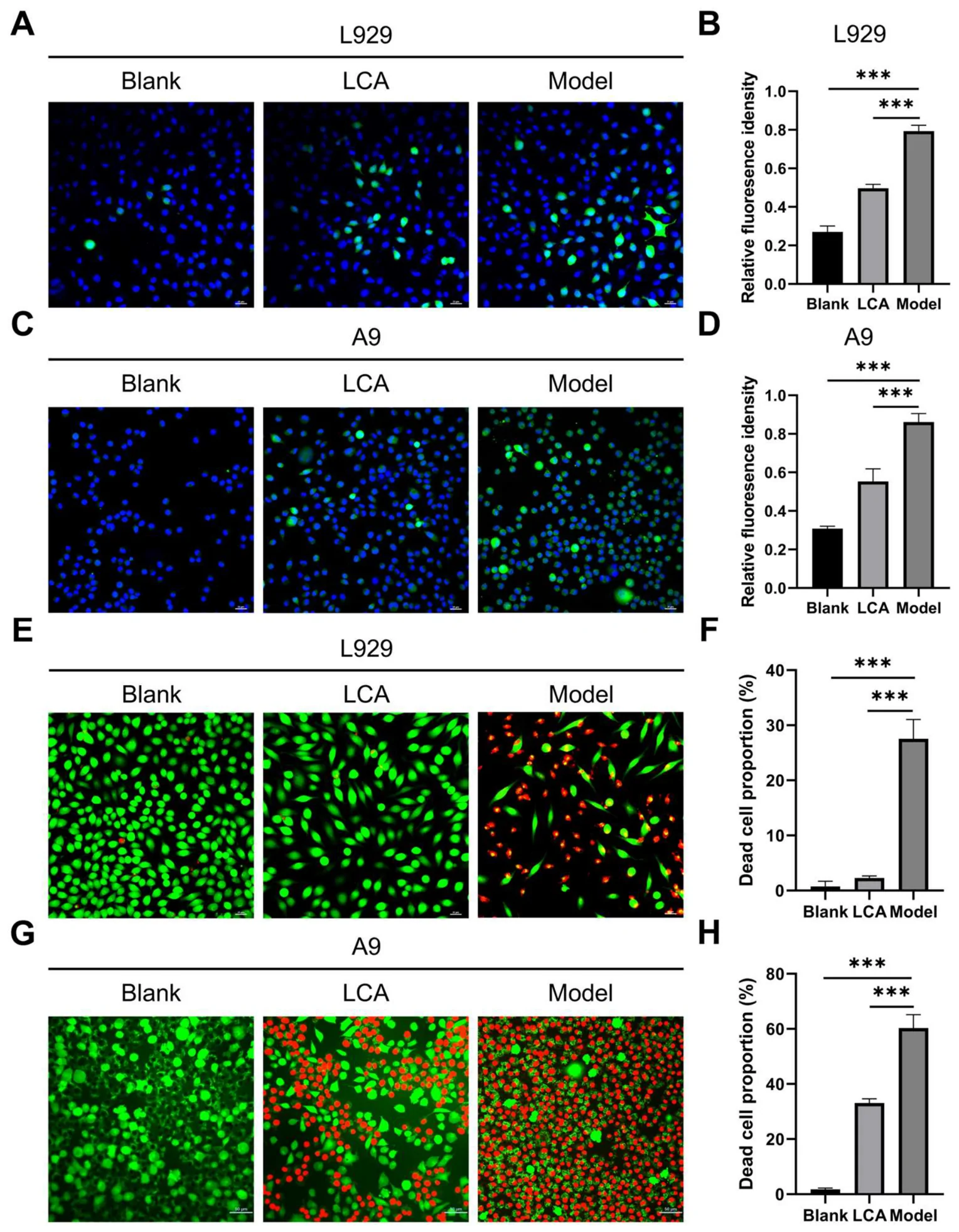
LCA reduces intracellular ROS accumulation and suppresses H_2_O_2_-triggered cell death in H_2_O_2_-stimulated fibroblasts. (**A**) Representative DCFH-DA fluorescence images of L929 cells (400× magnification); green fluorescence indicates ROS, and blue fluorescence indicates DAPI-stained nuclei. (**B**) Quantification of relative ROS fluorescence intensity in L929 cells. (**C**) Representative DCFH-DA fluorescence images of A9 cells (400× magnification); green fluorescence indicates ROS, and blue fluorescence indicates DAPI-stained nuclei. (**D**) Quantification of relative ROS fluorescence intensity in A9 cells. (**E**) Representative Calcein-AM/PI double-staining images of L929 cells (400× magnification); green fluorescence indicates live cells, and red fluorescence indicates dead cells. (**F**) Quantification of the dead cell proportion (%) in L929 cells. (**G**) Representative Calcein-AM/PI double-staining images of A9 cells (400× magnification); green fluorescence indicates live cells, and red fluorescence indicates dead cells. (**H**) Quantification of the dead cell proportion (%) in A9 cells. Data are expressed as mean ± SD. *** *p* < 0.001 vs. Model group.

Intracellular ROS levels were assessed by DCFH-DA fluorescence staining. H_2_O_2_ stimulation markedly increased the green fluorescence area and intensity in both L929 and A9 cells, whereas LCA pretreatment visibly attenuated the fluorescence signal (Figure 5A,C). Quantitative analysis confirmed that the relative ROS fluorescence intensity was significantly lower in the LCA group than in the Model group (both *p* < 0.001; Figure 5B,D), indicating effective ROS scavenging activity of LCA.

### 2.6. LCA Decreases H_2_O_2_-Induced Cell Death

Calcein-AM/PI double staining was used to distinguish live and dead cells. In the Model group, intense red fluorescence predominated, indicating that most cells had died following H_2_O_2_ stimulation. In contrast, the LCA group contained markedly fewer PI-positive dead cells and more calcein-positive live cells with intact morphology (Figure 5E,G). Quantitative analysis showed that the dead cell proportion was markedly elevated in the Model group relative to the Blank controls and was significantly reduced by LCA pretreatment in both L929 and A9 cells (both *p* < 0.001; Figure 5F,H), demonstrating that LCA protects fibroblasts from oxidative cell death.

### 2.7. LCA Preserves Fibroblast Migration Capacity

**Figure 6:**
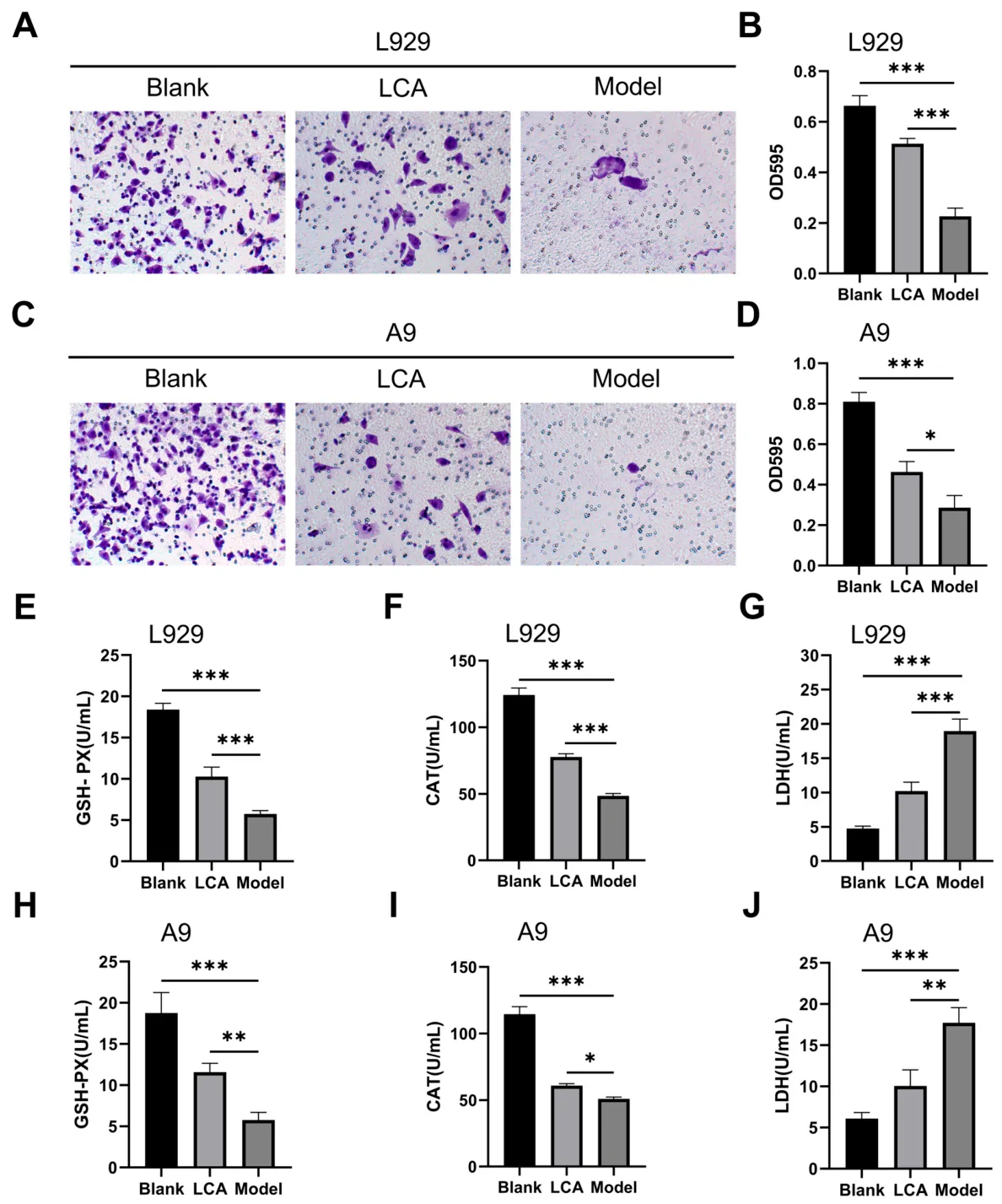
LCA preserves fibroblast migration capacity, restores antioxidant enzyme activities and alleviates oxidative damage in H_2_O_2_-stimulated fibroblasts. (**A**,**C**) Representative crystal violet-stained Transwell migration images of L929 (**A**) and A9 (**C**) cells (200× magnification). (**B**,**D**) Quantification of migrated L929 (**B**) and A9 (**D**) cells determined by crystal violet absorbance at 595 nm. (**E**–**G**) Levels of GSH-PX (**E**), CAT (**F**), and LDH (**G**) in culture supernatants of L929 cells. (**H**–**J**) Levels of GSH-PX (**H**), CAT (**I**), and LDH (**J**) in culture supernatants of A9 cells. Data are expressed as mean ± SD. * *p* < 0.05, ** *p* < 0.01, *** *p* < 0.001 vs. Model group.

Since fibroblast migration is a major determinant of burn wound-healing rate, Transwell assays were performed to evaluate cell migration after LCA pretreatment. The number of migrated cells was highest in the Blank group, followed by the LCA group, whereas migration was severely suppressed in the Model group, in which few cells with poor morphology traversed the membrane (Figure 6A,C). Quantification of crystal violet absorbance at 595 nm confirmed that the migration capacity of the LCA group was significantly greater than that of the Model group in both cell lines (*p* < 0.001; Figure 6B,D). These results suggest that LCA maintains fibroblast migratory capacity.

### 2.8. LCA Restores Antioxidant Enzyme Activities and Reduces Oxidative Damage

The oxidative stress indices GSH-PX, CAT, and LDH were measured in cell culture supernatants. In both L929 and A9 cells, GSH-PX and CAT levels were significantly higher in the LCA group than in the Model group, whereas LDH release was significantly lower, with the significance levels for each indicator as indicated in Figure 6E–J (* *p* < 0.05, ** *p* < 0.01, *** *p* < 0.001 vs. Model), indicating that LCA preserves H_2_O_2_-scavenging enzyme activities and alleviates oxidative membrane damage.

### 2.9. LCA Suppresses Inflammatory Cytokine Production in Oxidatively Stressed Fibroblasts

**Figure 7:**
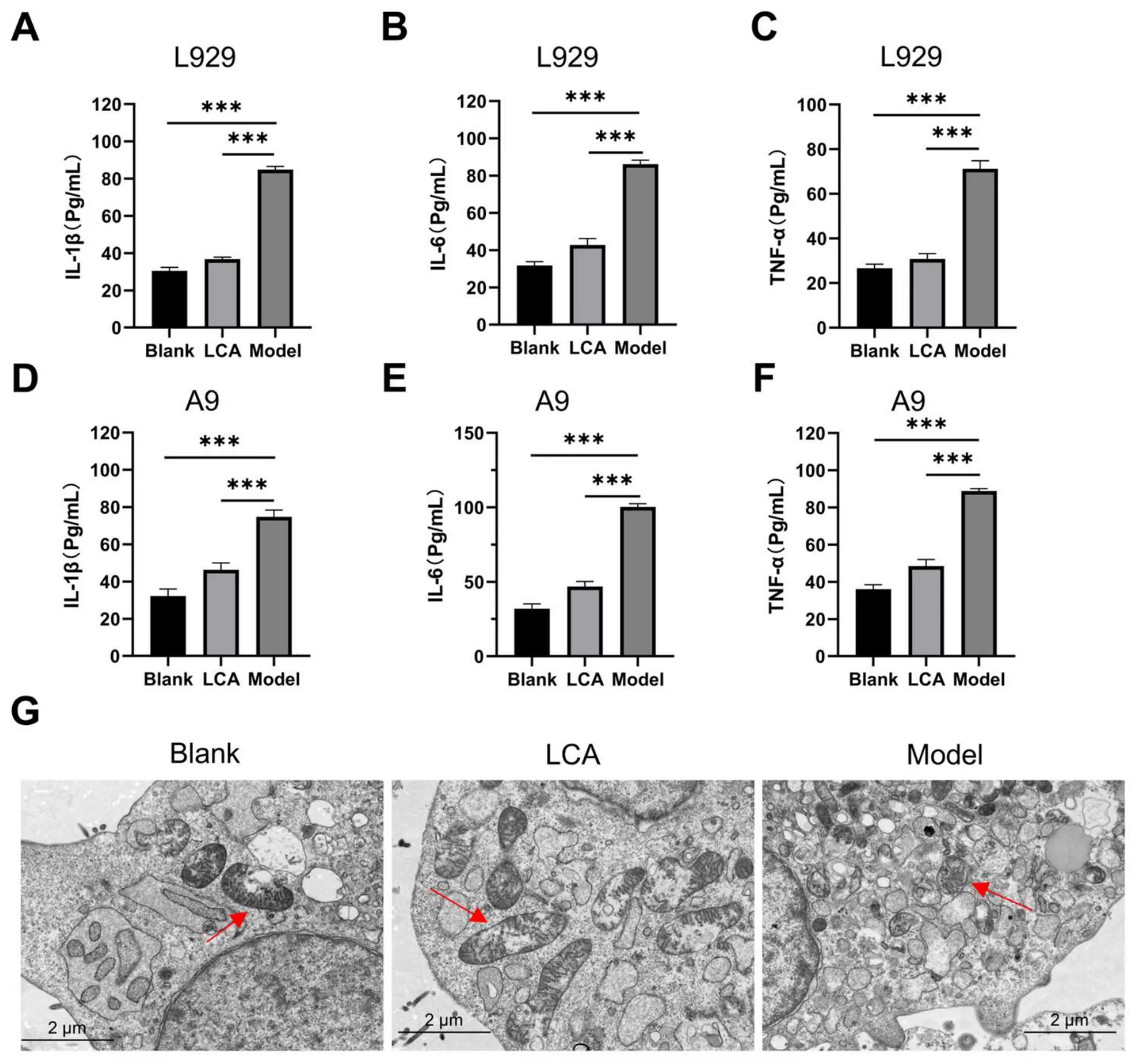
LCA inhibits inflammatory cytokine release and maintains mitochondrial ultrastructure in H_2_O_2_-stimulated fibroblasts. (**A**–**C**) ELISA-detected concentrations of IL-1β (**A**), IL-6 (**B**), and TNF-α (**C**) in L929 cell supernatants. (**D**–**F**) ELISA-detected concentrations of IL-1β (**D**), IL-6 (**E**), and TNF-α (**F**) in A9 cell supernatants. (**G**) Representative transmission electron microscopy images showing mitochondrial ultrastructure of L929 cells. Data are expressed as mean ± SD. *** *p* < 0.001, vs. Blank or Model group. The red arrow indicates the mitochondrion.

Consistent with the in vivo immunohistochemistry results, ELISA analysis showed that the concentrations of IL-1β, IL-6, and TNF-α in cell culture supernatants rose sharply after H_2_O_2_ stimulation (all *p* < 0.001 vs. Blank), and that LCA pretreatment significantly reduced the levels of IL-1β, IL-6, and TNF-α compared with the Model group (all *p* < 0.001, Figure 7A–F). These findings show that LCA attenuates the inflammatory response.

### 2.10. LCA Preserves Mitochondrial Ultrastructure

Transmission electron microscopy (TEM) was used to examine the ultrastructure of L929 cells. In the Blank group, mitochondria were intact and oval, with clearly visible cristae formed by infolding of the inner membrane. In the Model group, the mitochondrial architecture was severely disrupted, with fragmented organelles and widespread cellular debris. In the LCA group, the mitochondria remained largely intact and oval with discernible cristae (Figure 7G), demonstrating that LCA protects mitochondrial ultrastructure against oxidative injury.

### 2.11. Integrated Transcriptomic and Proteomic Analyses Identify Oxidative Stress–Related Pathways

**Figure 8:**
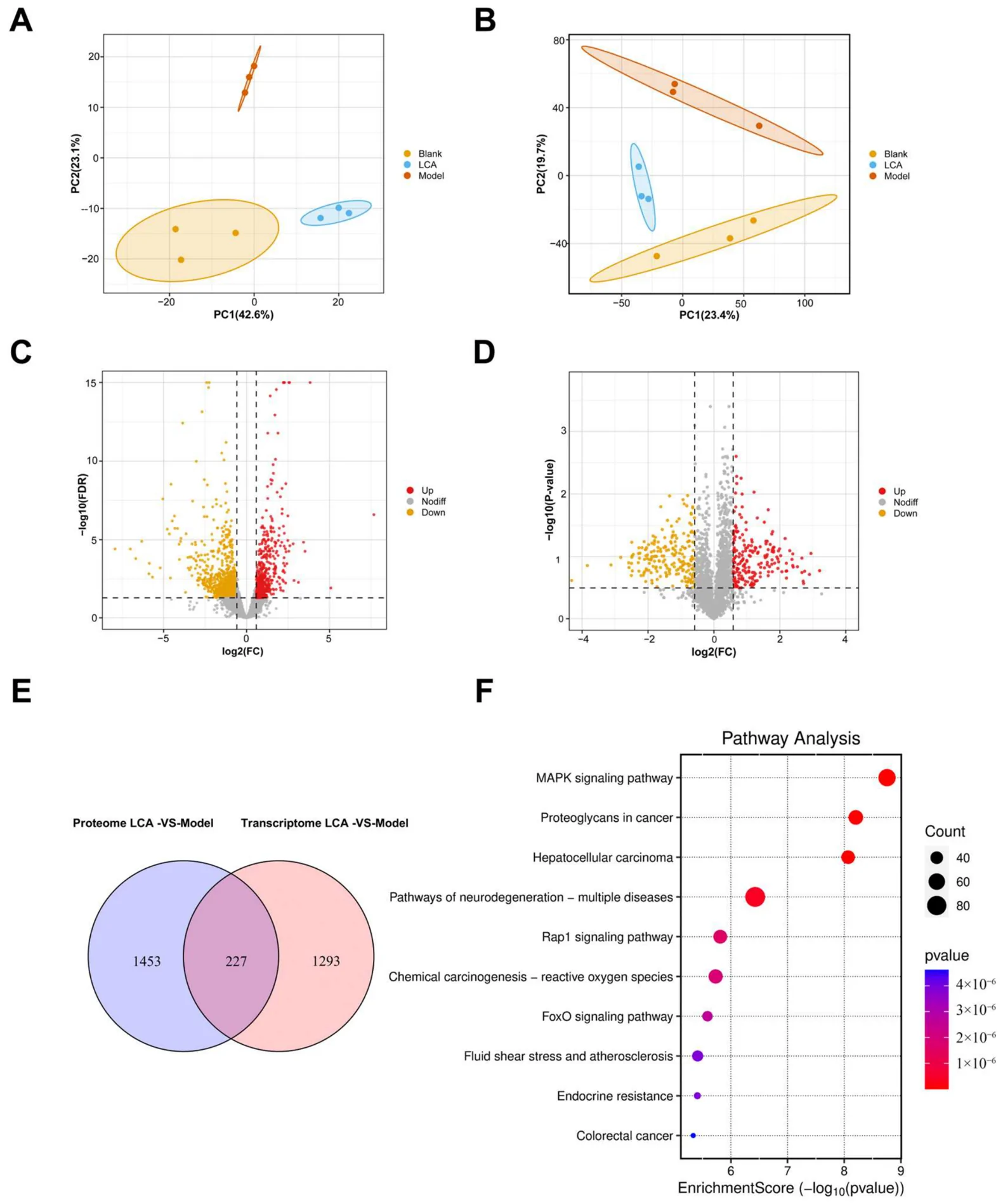
Integrated transcriptomic and proteomic analyses of burn wound skin (*n* = 3 per group). (**A**) PCA plot of the transcriptomic profiles. (**B**) PCA plot of the proteomic profiles. (**C**) Volcano plot of DEGs between the LCA and Model groups (693 upregulated and 826 downregulated). (**D**) Volcano plot of DEPs between the LCA and Model groups (966 upregulated and 914 downregulated). (**E**) Venn diagram of the 227 overlapping differentially expressed genes/proteins. (**F**) KEGG pathway enrichment analysis of the overlapping genes/proteins; ROS-related pathways are among the top 10 enriched pathways.

To further investigate the molecular mechanisms underlying the protective effects of LCA against burn-wound oxidative injury, integrated transcriptomic and proteomic analyses were performed on day-9 burn wound skin (*n* = 3 per group). As shown in Figure 8A,B, principal component analysis (PCA) demonstrated clear separation among the Blank, LCA and Model groups in both transcriptomic and proteomic datasets, indicating that burn injury and LCA treatment induced substantial changes in gene and protein expression profiles. Transcriptomic analysis identified 693 upregulated genes and 826 downregulated genes in the LCA group relative to the Model group (Figure 8C). Proteomic analysis identified 966 upregulated proteins and 914 downregulated proteins (Figure 8D). Integration of the two datasets revealed 227 overlapping molecules that were significantly altered at both the mRNA and protein levels, including 101 upregulated and 126 downregulated genes/proteins (Figure 8E). Kyoto Encyclopedia of Genes and Genomes (KEGG) pathway enrichment analysis of these overlapping molecules showed significant enrichment in several pathways, among which the chemical carcinogenesis-reactive oxygen species pathway and the MAPK signaling pathway were most closely associated with oxidative-stress-mediated tissue injury (Figure 8F). These findings indicate that LCA induces broad transcriptomic and proteomic alterations in burn wound skin.

### 2.12. LCA Modulates the Keap1/Nrf2/HO-1 Antioxidant Pathway and Restores NQO1 and GPX4 Expression

**Figure 9:**
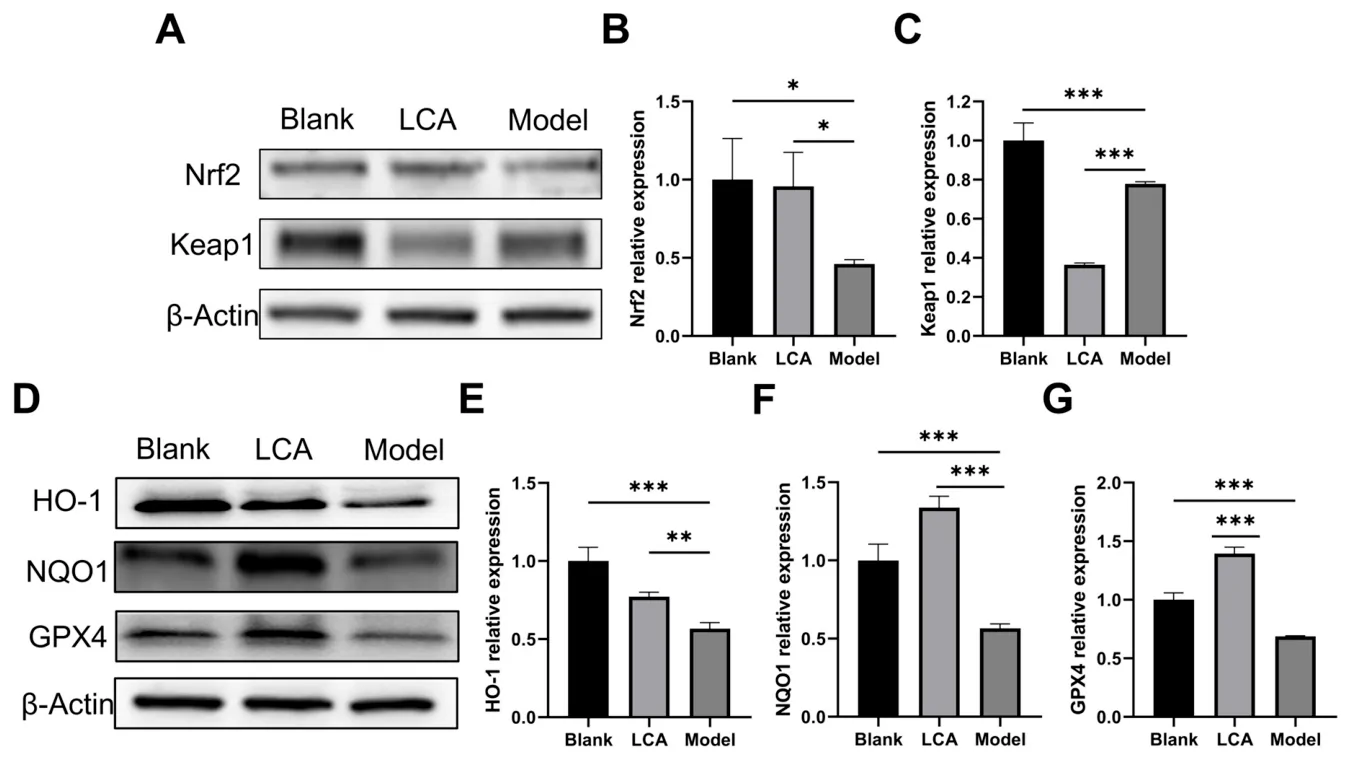
LCA modulates the Keap1/Nrf2/HO-1 antioxidant pathway and restores NQO1 and GPX4 expression in H_2_O_2_-stimulated fibroblasts. (**A**) Representative Western blot images of Nrf2 and Keap1. (**B**) Quantification of Nrf2 relative expression normalized to β-actin. (**C**) Quantification of Keap1 relative expression normalized to β-actin. (**D**) Representative Western blot images of HO-1, NQO1, and GPX4. (**E**) Quantification of HO-1 relative expression normalized to β-actin. (**F**) Quantification of NQO1 relative expression normalized to β-actin. (**G**) Quantification of GPX4 relative expression normalized to β-actin. Fibroblasts were pretreated with LCA for 24 h and then stimulated with H_2_O_2_ for 12 h. Data are expressed as mean ± SD (*n* = 3); * *p* < 0.05, ** *p* < 0.01, *** *p* < 0.001 (comparisons between the indicated groups).

Western blot analysis was performed to evaluate the effects of LCA on Keap1/Nrf2/HO-1 pathway-related protein expression in fibroblasts. H_2_O_2_ stimulation significantly downregulated Nrf2 (*p* < 0.05 vs. Blank), Keap1 (*p* < 0.001 vs. Blank), and the downstream effectors HO-1, NQO1, and GPX4 (all *p* < 0.001 vs. Blank). LCA pretreatment significantly restored the expression of Nrf2 (*p* < 0.05 vs. Model), HO-1 (*p* < 0.01 vs. Model), NQO1 (*p* < 0.05 vs. Model), and GPX4 (*p* < 0.001 vs. Model), and further decreased Keap1 expression (*p* < 0.001 vs. Model) (Figure 9A–G), consistent with enhanced Nrf2 accumulation upon downregulation of its negative regulator Keap1. These results indicate that LCA treatment was associated with modulation of the Keap1/Nrf2/HO-1 antioxidant pathway and restored expression of the Nrf2 target genes NQO1 and GPX4, consistent with enhanced cellular capacity to scavenge ROS and counteract lipid peroxidation.

## 3. Discussion

Second-degree burn wound healing remains a significant clinical challenge, and the identification of natural bioactive compounds with wound-healing potential is of considerable translational interest. In this exploratory study, we observed that a topical LCA-containing hydrogel (LCA-hydrogel) was associated with accelerated second-degree burn wound healing in mice, accompanied by attenuated inflammation and oxidative stress, as well as enhanced collagen deposition, cell proliferation, and angiogenesis. Integrating in vivo, in vitro, transcriptomic, and proteomic evidence, our data suggest that the antioxidant effects of LCA are associated with modulation of Keap1/Nrf2/HO-1 signaling and of the downstream effectors NQO1 and GPX4.

The inflammatory response after burn injury is a double-edged sword: moderate inflammation is essential for debridement and defense against infection, whereas excessive or persistent inflammation amplifies tissue damage and delays healing. In the present study, LCA-hydrogel treatment was associated with reduced expression of pro-inflammatory cytokines and the infiltration of neutrophils and macrophages in wound tissue, and these anti-inflammatory effects were recapitulated in H_2_O_2_-stimulated fibroblasts, which is compatible with a direct action of LCA on resident skin cells as well as infiltrating immune cells. These observations are consistent with the well-documented anti-inflammatory properties of LCA, including suppression of the MAPK and NF-κB pathways in macrophages and microglia [17,18], and with the anti-inflammatory activity of licorice constituents reported in other systems [24,25]. By restraining excessive inflammation and promoting collagen deposition, proliferation, and angiogenesis, the LCA-hydrogel may create a microenvironment favorable for tissue repair.

Oxidative stress is a key driver of impaired burn wound healing, because excessive ROS damage lipids, proteins, and DNA and suppress fibroblast proliferation and migration. In this study, LCA lowered intracellular ROS, restored the activities of the H_2_O_2_-scavenging enzymes GSH-PX and CAT, reduced LDH release, and preserved mitochondrial ultrastructure in H_2_O_2_-stimulated fibroblasts, consistent with the protective effects of LCA reported in other oxidative-stress-related injury models [20,21,23]. At the molecular level, integrated transcriptomic and proteomic analysis identified the chemical carcinogenesis–reactive oxygen species pathway as the pathway most relevant to the effects of the LCA-hydrogel, and Western blotting showed lower Keap1 and higher Nrf2, HO-1, NQO1, and GPX4 expression after LCA treatment, indicating that LCA treatment was associated with modulation of Keap1/Nrf2/HO-1 signaling. Nrf2 is known to accumulate and induce antioxidant genes such as HO-1 [26,27]; the concurrent restoration of NQO1 and GPX4, two known Nrf2 target genes, is compatible with Nrf2-related antioxidant defense, in line with genetic evidence that Nrf2 is essential for cutaneous wound repair [28]. Notably, extract of Abelmoschus manihot, applied to a comparable deep second-degree scald model, similarly attenuated oxidative markers and modulated Nrf2 expression, though without concurrent transcriptomic/proteomic profiling [29]. Our integration of multi-omics data extends this line of evidence by showing that HO-1, NQO1, and GPX4 expression changed together with Nrf2 and Keap1 after LCA treatment. Curcumin has been reported to activate the Nrf2/HO-1 axis to promote full-thickness skin wound healing, with a distinct downstream program centered on caspase-3-mediated apoptosis rather than ferroptosis-related effectors [30]. In contrast, quercetin, a flavonoid derived from a different medicinal plant, promotes cutaneous wound healing predominantly via Wnt/β-catenin signaling rather than Nrf2 signaling [31], indicating that the wound-healing benefits of flavonoids are not mediated by a single conserved pathway. Although the present findings do not establish a causal mechanism, the observed changes in Keap1/Nrf2/HO-1 and its downstream targets suggest an association between LCA treatment and modulation of this pathway. Our results thus extend the pharmacology of LCA to burn wound healing and suggest that LCA, delivered in a hydrogel, may be a candidate for topical burn therapy. Several currently used burn treatments help illustrate where the LCA-hydrogel differs. Silver sulfadiazine is cytotoxic to keratinocytes and fibroblasts and delays re-epithelialization [8,9], and silver-containing formulations have also been reported to impair neovascularization compared with other topical agents [9]; the LCA-hydrogel, in this study, was associated with increased Ki67 and VEGF-C expression in wound tissue (Figure 3E–H), consistent with a possible role in proliferation and angiogenesis rather than antimicrobial action alone. Surgical excision and skin grafting remain constrained by donor-site morbidity [10,11], while the LCA-hydrogel is non-invasive and simple to apply. Hydrocolloid and other advanced dressings are costly, need frequent replacement, and function mainly as passive barriers [12]; the LCA-hydrogel instead pairs moisture retention with intrinsic antioxidant, anti-inflammatory, and pro-angiogenic activity. As a plant-derived flavonoid, LCA also carries the general advantage of multi-target activity, which sets it apart from single-mechanism synthetic agents such as silver sulfadiazine [13,14].

This study has several limitations: no vehicle-only hydrogel group was included, and the Model group received no treatment. The in vivo effects therefore cannot be attributed to LCA alone, because they may also reflect the carbomer base, wound hydration, coverage, or the daily application itself, and the results are best viewed as effects of the LCA-containing hydrogel; only female mice were used, since male mice fought and confounded wound assessment, so the findings may not generalize to males, given known sex differences in healing; wound closure was assessed in different mice at each time point and compared between groups separately at each time point, without modeling the treatment × time interaction, so closure kinetics should be interpreted descriptively; outcome assessment was not fully blinded, which may introduce observer bias into the measurement of wound area and the histological and immunohistochemical quantification and may overestimate the effects; Nrf2/HO-1 expression was assessed only at the protein level in cultured fibroblasts, without nuclear translocation, pathway inhibition, ferroptosis markers, or knockdown/rescue experiments, so these findings remain associative and do not establish a causal role for Keap1/Nrf2/HO-1 signaling in wound healing; the omics analyses used only three samples per group, which limits statistical power and the reproducibility of the identified genes and pathways, and the findings were not independently validated in wound tissue; the raw omics data have not been deposited in a public repository, which limits independent verification and reanalysis; only a single 1% LCA dose was tested, selected from preliminary dose-finding observations that cannot substitute for a formal dose–response experiment, so no dose–response relationship, optimal dose, or safety margin has been established; the in vitro working concentrations were close to the cytotoxic range (particularly 30 μM in A9 cells), which limits the interpretation of the protective and functional assays, and confirmation at lower, non-cytotoxic concentrations is required; the comparisons above with silver sulfadiazine, skin grafting, and advanced dressings are literature-based rather than head-to-head, warranting future comparative studies. Finally, the many endpoints assessed were not corrected for multiplicity across analyses (Tukey’s test was applied only to pairwise comparisons within each analysis). These analyses should therefore be regarded as exploratory, the risk of Type I error is increased, and the conclusions should be interpreted with caution and confirmed in independent studies.

## 4. Materials and Methods

### 4.1. Reagents and Cell Culture

Licochalcone A (Cat. No. B20409; purity ≥ 98%, Figure 1A) was purchased from Shanghai Yuanye Biotechnology (Shanghai, China). Moist exposed burn ointment (MEBO; National medicine approval number: Z20000004) was obtained from Shantou Meibo Pharmaceutical Co., Ltd. (Shantou, China). Carbomer 940 (Cat. No. C299587), glycerol (Cat. No. G116208), and triethanolamine (Cat. No. T108153) were purchased from Shanghai Aladdin Biochemical Technology (Shanghai, China). Hematoxylin (Cat. No. G1004), eosin (Cat. No. G1001), differentiation solution (Cat. No. G1039), bluing solution (Cat. No. G1040), Sirius red staining kits (Cat. No. G1078), Masson’s trichrome staining kits (Cat. No. G1006), and the CCK-8 cell counting kit (Cat. No. G4103) were obtained from Servicebio (Wuhan, China). Neutral balsam (Cat. No. G8590) and dimethyl sulfoxide (DMSO; Cat. No. D8371) were obtained from Solarbio (Beijing, China). The universal mouse/rabbit immunohistochemistry secondary antibody kit (Cat. No. abs996) and the Calcein-AM/PI live/dead cell double-staining kit (Cat. No. abs50056) were purchased from Absin (Shanghai, China). The reactive oxygen species (ROS) assay kit (DCFH-DA) (Cat. No. CA1410) and basement membrane matrix (Matrigel) (Cat. No. M8370) were obtained from Solarbio (Beijing, China). Glutathione peroxidase (GSH-PX; Cat. No. A005-1-2), catalase (CAT; Cat. No. A007-1-1), and lactate dehydrogenase (LDH; Cat. No. A020-2-2) assay kits were obtained from Nanjing Jiancheng Bioengineering Institute (Nanjing, China). Mouse interleukin-1β (IL-1β; Cat. No. ml098416), interleukin-6 (IL-6; Cat. No. ml098430), and tumor necrosis factor-α (TNF-α; Cat. No. ml002095) enzyme-linked immunosorbent assay (ELISA) kits were purchased from Shanghai Enzyme-Linked Biotechnology (Shanghai, China). Mouse fibroblast L929 cells (Cat. No. CL-0137) and mouse subcutaneous connective tissue A9 (Cat. No. CL-0018) cells were provided by Procell (Wuhan, China). L929 cells were cultured in Minimum Essential Medium (MEM; Cat. No. PM150410), whereas A9 cells were maintained in Dulbecco’s Modified Eagle Medium (DMEM; Cat. No. PM150210). Both media were supplemented with 10% fetal bovine serum (FBS; Cat. No. 164210) and 1% penicillin-streptomycin (P/S; Cat. No. PB180120). Cells were incubated at 37 °C in a humidified atmosphere containing 5% CO_2_, and cells within ten passages were used for all experiments. For in vitro experiments, LCA was dissolved in DMSO to prepare stock solutions and diluted with culture medium before use. The final DMSO concentration was identical across all treatment groups and did not exceed 0.1% (*v*/*v*).

### 4.2. Preparation of the LCA Hydrogel

The LCA hydrogel was prepared using a carbomer 940-based formulation. Briefly, carbomer 940 (1.2%, *w*/*w*) was swollen in distilled water overnight, glycerol (10%, *w*/*w*) and LCA (final concentration 1%, *w*/*w*) were added with continuous stirring, and triethanolamine (1.5%, *w*/*w*) was added dropwise to adjust the pH to 6.5–7.5 and induce gelation. The resulting hydrogel exhibited good spreadability, physical stability under high- and low-temperature storage, and compliance with the requirements for hydrogels in the Chinese Pharmacopoeia; this formulation was used for all topical treatments. The 1% LCA concentration was selected based on preliminary dose-finding experiments, in which this concentration produced wound healing in preliminary experiments, with no visible signs of local irritation; this concentration is also consistent with topical LCA hydrogel formulations reported to be biologically safe in murine wound-healing models [32].

### 4.3. Mouse Model and Topical Treatment

A total of 90 specific pathogen-free (SPF) female C57BL/6J mice (6–8 weeks old, 24–26 g) were purchased from Sipeifu Biotechnology Co., Ltd. (Beijing, China) and used in three cohorts: a main cohort (*n* = 45), a MEBO comparator cohort (*n* = 36), and an omics cohort (*n* = 9). Mice were housed at the Laboratory Animal Center of Tarim University under a 12 h light/12 h dark cycle at 25 ± 0.5 °C and 55 ± 2% relative humidity, with free access to food and water. All mice were acclimatized for 5 days before the experiments. The 45 mice of the main cohort were randomly allocated to the Blank, Model, and LCA groups (*n* = 15/group) by a single investigator prior to intervention: (1) Blank group: normal mice without any treatment; (2) Model group: second-degree thermal burn induced on the dorsal skin without treatment; (3) LCA group: second-degree thermal burn induced on the dorsal skin followed by topical application of the LCA hydrogel. For burn induction, the dorsal hair was removed with 6% sodium sulfide solution, mice were anesthetized by intraperitoneal injection of sodium pentobarbital (50 mg/kg), and a second-degree thermal burn was induced on the dorsal skin using a self-made temperature-controlled burning instrument at 100 °C for 5 s (Supplement Video S1). Mice in the LCA group received topical application of approximately 0.2 g of the LCA hydrogel (≈2 mg LCA per wound) to the burn wound once daily from day 1. This application volume was determined empirically to ensure complete and even coverage of the wound surface while maintaining hydrogel hydration, and corresponds to a total LCA dose (~2 mg) consistent with the concentration selected in Section 4.2. Mice in the Blank and Model groups were left untreated. Each group comprised five time-point subgroups (days 1, 3, 5, 7, and 9 after burn induction; *n* = 3 mice per group per time point). At each time point, the mice of the corresponding subgroup were euthanized by cervical dislocation, and full-thickness wound skin together with approximately 0.3 cm of surrounding tissue was excised under aseptic conditions and fixed in 4% paraformaldehyde for 24 h for histological analyses. Day 9 was the prespecified end of the experiment, and all histological and immunohistochemical analyses reported in this study were performed on day-9 samples (*n* = 3 mice per group). To benchmark wound-closure kinetics against a clinically used comparator, a separate cohort (*n* = 36) was prepared in an independent experiment, comprising the Model, LCA (treated as described above), and MEBO groups with *n* = 3 mice per group at each of days 3, 5, 7, and 9. MEBO ointment was applied once daily from day 1 following the same burn-induction protocol described above, and wound closure was photographed and quantified at each time point (Supplementary Figure S1). For transcriptomic and proteomic analyses, wound skin samples were collected on day 9 from a separate cohort of identically treated mice (*n* = 3 each in the Blank, Model, and LCA groups) and immediately frozen in liquid nitrogen. Mice in the MEBO comparator and omics cohorts were randomly allocated to their groups in the same manner as in the main cohort.

### 4.4. Sirius Red and Masson’s Trichrome Staining

Fixed skin samples were dehydrated through a graded ethanol series, cleared in xylene, embedded in paraffin, and sectioned. For Sirius red staining, deparaffinized sections were stained with Sirius red solution for 8 min, dehydrated in absolute ethanol, cleared in xylene, and mounted with neutral balsam. For Masson’s trichrome staining, sections were stained sequentially with Weigert’s iron hematoxylin for 5 min, differentiated in acidic ethanol, stained with Ponceau–acid fuchsin for 5 min, treated with 1% phosphomolybdic acid for 5 min, counterstained with aniline blue for 5 min, dehydrated, cleared, and mounted. Collagen deposition was observed by light microscopy, and the relative collagen content was quantified using ImageJ software (version 1.53). For histological quantification, three mice per group (day-9 samples) were used; three sections per mouse were analyzed, with five non-overlapping fields per section quantified using ImageJ.

### 4.5. Immunohistochemical Staining

Paraffin sections of wound skin collected on day 9 were deparaffinized, rehydrated, and subjected to antigen retrieval in sodium citrate buffer. After quenching of endogenous peroxidase activity, sections were blocked with 5% goat serum for 20 min at room temperature and incubated with primary antibodies against IL-1β (Cat. No. bsm-55644M, 1:500), IL-6 (Cat. No. bsm-10807M, 1:500), TNF-α (Cat. No. bsm-55603R, 1:500), Ly6G (Cat. No. bsm-30178A, 1:500), F4/80 (Cat. No. bs-7058R, 1:400), Ki67 (Cat. No. bsm-52455R, 1:500), and vascular endothelial growth factor C (VEGF-C; Cat. No. bs-20740R, 1:400; all from Bioss, Beijing, China) at 37 °C for 30 min, followed by incubation with the universal secondary antibody kit (Absin, Shanghai, China). 3,3′-Diaminobenzidine (DAB) was used as the chromogen, and nuclei were counterstained with hematoxylin. Images were captured by light microscopy, and positive staining was quantified using ImageJ software. For each immunohistochemical marker, three mice per group (day-9 samples) were analyzed; three sections per mouse were examined, with five fields per section quantified for positive staining area/cell counts.

### 4.6. Cell Viability Assay and Establishment of the Oxidative Stress Model

Cell viability was measured using the CCK-8 assay. L929 and A9 cells were seeded in 96-well plates at 5 × 10^3^ cells/well and cultured for 24 h. For cytotoxicity assessment, cells were treated with LCA at 0, 10, 15, 20, 25, 30, 35, or 40 μM for 24 h. To establish the oxidative stress injury model, cells were stimulated with H_2_O_2_ at 0, 400, 600, 800, 1000, 1200, or 1400 μM for 12 h. For protection assays, cells were pretreated with LCA (20 μM for L929 cells; 30 μM for A9 cells) for 24 h and then exposed to H_2_O_2_ (1000 μM for L929 cells; 1200 μM for A9 cells) for 12 h. CCK-8 solution was then added to each well, and after incubation for 1 h at 37 °C, the absorbance at 450 nm was recorded with a microplate reader (K6600A+, Kaiao, Beijing, China).

Cell viability (%) = [A(treated) − A(blank)]/[A(untreated) − A(blank)] × 100.

### 4.7. Intracellular ROS Detection

Intracellular ROS levels were determined using the fluorescent probe 2′,7′-dichlorodihydrofluorescein diacetate (DCFH-DA). L929 and A9 cells were seeded at 2 × 10^5^ cells/well in 6-well plates and cultured for 24 h. Cells were then pretreated with LCA (20 μM for L929 cells; 30 μM for A9 cells) for 24 h and exposed to H_2_O_2_ (1000 μM for L929 cells; 1200 μM for A9 cells) for 12 h. After the treatments, cells were incubated with DCFH-DA working solution (diluted 1:1000 in serum-free medium) for 30 min at 37 °C in the dark, washed three times with phosphate-buffered saline (PBS), counterstained with DAPI for 5 min at 37 °C, and washed again. Fluorescence images were captured with an inverted fluorescence microscope (Zeiss, Oberkochen, Germany), and the relative fluorescence intensity was quantified using ImageJ software.

### 4.8. Calcein-AM/PI Live/Dead Cell Staining

Live and dead cells were distinguished using the Calcein-AM/propidium iodide (PI) double-staining kit (Absin, Shanghai, China). L929 and A9 cells were seeded at 2 × 10^5^ cells/well in 6-well plates and cultured for 24 h, pretreated with LCA (20 μM for L929 cells; 30 μM for A9 cells) for 24 h, and then exposed to H_2_O_2_ (1000 μM for L929 cells; 1200 μM for A9 cells) for 12 h. Calcein-AM is converted by intracellular esterases into green fluorescent calcein in live cells, whereas PI penetrates only compromised membranes and stains dead cells red. After the treatments, cells were washed with 1× Assay Buffer and incubated with the working solution for 15 min at 37 °C, and fluorescence images were captured by fluorescence microscopy.

### 4.9. Transwell Migration Assay

Cell migration was evaluated using Transwell chambers. The upper chambers were coated with 60 μL of basement membrane matrix diluted 8:1 in the corresponding serum-free medium (MEM for L929 cells; DMEM for A9 cells) and allowed to solidify at 37 °C for 30 min. Cell suspensions (200 μL containing 5 × 10^4^ cells) were added to the upper chambers, and complete medium containing 20% FBS (650 μL) was added to the lower chambers. Cells were then treated according to the oxidative stress injury model: pretreated with LCA (20 μM for L929 cells; 30 μM for A9 cells) for 24 h, followed by exposure to H_2_O_2_ (1000 μM for L929 cells; 1200 μM for A9 cells) for 12 h. After 36 h at 37 °C in 5% CO_2_, cells on the upper side of the membrane were gently removed with cotton swabs, and migrated cells on the lower side were fixed with 4% paraformaldehyde for 20 min and stained with 0.1% crystal violet for 30 min. Images were captured by microscopy at 200× magnification. For quantification, crystal violet was dissolved in absolute ethanol and the absorbance was measured at 595 nm.

### 4.10. Oxidative Stress Indices and Inflammatory Cytokine Assays

Cell culture supernatants were collected by centrifugation at 3000 rpm for 15 min. The levels of GSH-PX, CAT, and LDH were determined using commercial assay kits (Nanjing Jiancheng Bioengineering Institute, Nanjing, China) according to the manufacturer’s instructions; CAT activity was calculated from the absorbance at 405 nm. The concentrations of IL-1β, IL-6, and TNF-α were measured using double-antibody sandwich ELISA kits (Shanghai Enzyme-Linked Biotechnology, Shanghai, China) according to the manufacturer’s instructions; absorbance was read at 450 nm, and sample concentrations were calculated from standard curves generated with GraphPad Prism 8.0.

### 4.11. Transmission Electron Microscopy

Cell pellets were immersed in 2.5% glutaraldehyde prepared in 0.1 M cacodylate buffer for 2 h at 4 °C and then post-fixed with 1% osmium tetroxide. Following stepwise dehydration in increasing ethanol concentrations (30–100%) and a final rinse in acetone, the specimens were embedded in Epon resin. Sections of approximately 70 nm in thickness were counterstained with uranyl acetate and lead citrate and imaged on a Hitachi High-Tech transmission electron microscope (Tokyo, Japan). Mitochondrial morphology was evaluated by an investigator unaware of the group assignments. TEM morphological assessment was performed by an observer blinded to group allocation. For wound assessment, histological quantification, immunohistochemical analysis, and Western blot quantification, formal blinding was not implemented; quantification relied on software-based measurements (ImageJ) rather than subjective scoring.

### 4.12. Transcriptome Sequencing and Analysis

Day-9 wound skin samples (*n* = 3 per group) were used for transcriptome sequencing. RNA was isolated with TransZol Up reagent (TransGen Biotech, Beijing, China). The integrity of the obtained RNA was verified on 1.5% agarose gels, and its quantity and purity were measured on a NanoDrop spectrophotometer. Polyadenylated mRNA was captured with Oligo(dT) magnetic beads (Thermo Fisher Scientific, Waltham, MA, USA); libraries were prepared with the VAHTS Universal V8 RNA-seq Library Prep Kit for Illumina (Vazyme, Nanjing, China) and run on an Illumina sequencer. Transcriptomic and proteomic profiling were performed on RNA and protein extracted from the same batch of day-9 wound skin samples; within each platform, all samples were processed and sequenced/analyzed as a single batch, precluding the need for batch-effect correction. Raw sequencing reads were quality-filtered using fastp (v0.20.1) with default parameters to remove adapter sequences and low-quality bases prior to alignment. Clean reads were aligned to the reference genome with STAR (v2.7.7a), and gene expression was quantified with featureCounts (v2.0.0) and StringTie (v2.2.1). Differential expression analysis was performed with edgeR (v3.42.4) using trimmed mean of M-values (TMM) normalization, and differentially expressed genes (DEGs) were defined by a false discovery rate (FDR) < 0.05 and |log_2_(fold change)| ≥ 1. Gene Ontology (GO) and Kyoto Encyclopedia of Genes and Genomes (KEGG) enrichment analyses were performed with clusterProfiler (v4), and corrected *p* ≤ 0.05 was considered significantly enriched.

### 4.13. Proteomic Analysis

Day-9 wound skin samples (*n* = 3 per group) were rinsed with ice-cold PBS, cut into small pieces, and homogenized on ice in lysis buffer containing protease inhibitors (tissue:buffer = 1:5–1:10, *w*/*v*). Homogenates were incubated on ice for 30 min and centrifuged at 12,000× *g* for 15 min at 4 °C, and protein concentrations of the supernatants were determined by bicinchoninic acid (BCA) assay. Proteins were digested into peptides with trypsin, desalted on Strata X C18 solid-phase extraction columns (Phenomenex, Torrance, CA, USA), and subjected to label-free quantitative proteomic analysis by liquid chromatography–tandem mass spectrometry (LC-MS/MS) in data-dependent acquisition (DDA) mode on an EASY-nLC 1000 system (Thermo Fisher Scientific) coupled to an Orbitrap Fusion mass spectrometer (Thermo Fisher Scientific) equipped with a ZORBAX 300Extend-C18 column (Agilent, Santa Clara, CA, USA). For each pairwise comparison, fold change (FC) was calculated as the ratio of mean relative quantification values between groups; relative quantification values were log2-transformed and analyzed with limma (v3.46.0) to obtain *p*-values. Differentially expressed proteins (DEPs) were defined as FC ≥ 1.5 and *p* < 0.05 (upregulated) or FC ≤ 0.667 and *p* < 0.05 (downregulated). GO and KEGG enrichment analyses of DEPs were performed using Fisher’s exact test, with a corrected *p* < 0.05 considered significantly enriched.

### 4.14. Western Blot Analysis

The primary antibodies comprised rabbit antibodies against Nrf2 (80593-1-RR), Keap1 (10503-2-AP), HO-1 (10701-1-AP), and NQO1 (11451-1-AP), each applied at 1:1000, together with β-actin (81115-1-RR; 1:5000) as the loading control, all supplied by Proteintech (Wuhan, China); GPX4 was detected using an antibody from Servicebio (GB124327; 1:1000; Wuhan, China). After rinsing, the membranes were probed for 1 h at ambient temperature with HRP-linked secondary antibodies—goat anti-rabbit IgG-HRP (bs-0295G-HRP) or goat anti-mouse IgG-HRP (bs-0296G-HRP), both at 1:5000 and obtained from Bioss (Beijing, China). Immunoreactive bands were visualized by enhanced chemiluminescence, with the exposure of each membrane individually adjusted to keep the signals within the linear range and avoid saturation. Densitometric analysis was carried out in ImageJ, using β-actin as the internal reference. Each Western blot experiment was independently repeated three times (*n* = 3).

### 4.15. Statistical Analysis

Values throughout this study are given as the mean ± standard deviation (SD). GraphPad Prism (version 8.0; GraphPad Software, San Diego, CA, USA) was used for all statistical evaluations. For comparisons involving more than two groups, one-way analysis of variance (ANOVA) was applied, followed by Tukey’s post hoc test for pairwise comparisons. Differences were regarded as statistically significant at *p* < 0.05, indicated in the figures as * *p* < 0.05, ** *p* < 0.01, and *** *p* < 0.001. Wound areas were compared among groups separately at each time point (one-way ANOVA followed by Tukey’s post hoc test). Because the mice assessed at different time points were different animals from the same batch and each mouse was measured only once, the data were not analyzed as repeated measurements, and the treatment × time interaction was not modeled; these comparisons are therefore exploratory.

## 5. Conclusions

In conclusion, topical application of an LCA-containing hydrogel was associated with accelerated second-degree burn wound healing in mice. LCA-hydrogel treatment was associated with increased collagen deposition, suppressed IL-1β, IL-6 and TNF-α expression and Ly6G^+^/F4/80^+^ inflammatory cell infiltration, and enhanced Ki67 and VEGF-C expression in vivo, while protecting fibroblasts from H_2_O_2_-induced oxidative injury in vitro by reducing ROS accumulation and cell death, preserving mitochondrial ultrastructure and migration capacity, and restoring antioxidant enzyme activities. Integrated transcriptomic and proteomic analyses, together with Western blot results, suggest that these effects are associated with modulation of Keap1/Nrf2/HO-1 signaling, including restored expression of the downstream effectors NQO1 and GPX4. These exploratory findings provide preliminary evidence for the LCA-containing hydrogel as a candidate for burn wound management and support its further preclinical evaluation.

## Data Availability

The original contributions presented in this study are included in the article/Supplementary Material. Further inquiries can be directed to the corresponding authors.

## Supplementary Materials

The following supporting information can be downloaded at https://www.mdpi.com/article/10.3390/ijms27219450/s1.

## Abbreviations

The following abbreviations are used in this manuscript:

BCA	Bicinchoninic Acid
CAT	Catalase
CCK-8	Cell Counting Kit-8
DAB	3,3′-Diaminobenzidine
DEG	Differentially Expressed Gene
DEP	Differentially Expressed Protein
DMEM	Dulbecco’s Modified Eagle Medium
DMSO	Dimethyl Sulfoxide
ECL	Enhanced Chemiluminescence
ELISA	Enzyme-Linked Immunosorbent Assay
FBS	Fetal Bovine Serum
GO	Gene Ontology
GPX4	Glutathione Peroxidase 4
GSH-PX	Glutathione Peroxidase
HO-1	Heme Oxygenase-1
HRP	Horseradish Peroxidase
IL-1β	Interleukin-1β
IL-6	Interleukin-6
Keap1	Kelch-like ECH-Associated Protein 1
KEGG	Kyoto Encyclopedia of Genes and Genomes
LCA	Licochalcone A
LDH	Lactate Dehydrogenase
MAPK	Mitogen-Activated Protein Kinase
MEM	Minimum Essential Medium
NF-κB	Nuclear Factor-κB
NQO1	NAD(P)H:Quinone Oxidoreductase 1
Nrf2	Nuclear Factor Erythroid 2-Related Factor 2
PBS	Phosphate-Buffered Saline
PCA	Principal Component Analysis
PVDF	Polyvinylidene Fluoride
P/S	Penicillin-Streptomycin
ROS	Reactive Oxygen Species
SPF	Specific Pathogen-Free
TEM	Transmission Electron Microscopy
TNF-α	Tumor Necrosis Factor-α
VEGF-C	Vascular Endothelial Growth Factor C
