# Supplementary material for: Licochalcone A Attenuates Oxidative Stress and Inflammation to Promote Second-Degree Burn Healing Through Nrf2/HO-1 Activation in Mice
Source: Int J Mol Sci. 2026 Oct 23;27(21):9450. doi: 10.3390/ijms27219450 (PMC13763448; doi:10.3390/ijms27219450)
Supplement: Supplementary file 1 [file ijms-27-09450-s001.zip › ijms-4566680-supplementary.pdf]

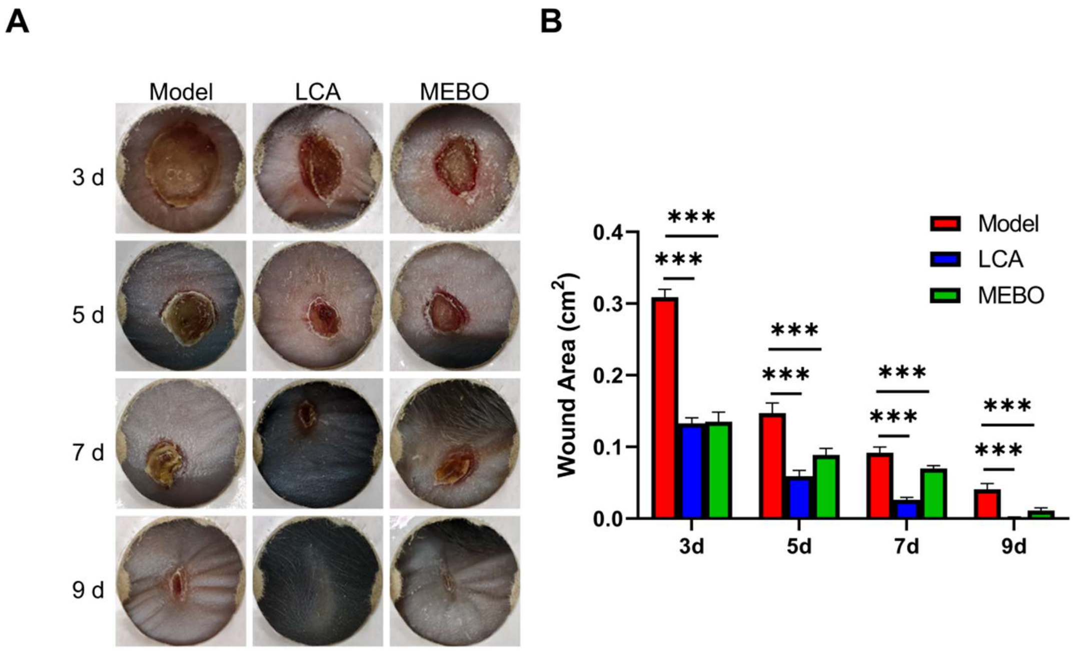

Figure S1. Wound area of Model, LCA, and MEBO groups in a murine second-degree burn model. (A) Representative photographs of wounds in the Model, LCA, and MEBO groups at days 3, 5, 7, and 9 post-burn. (B) Quantification of wound area (cm²) at each time point. Wound area was measured using ImageJ software, with the scale calibrated against the known 1 cm diameter of the fixation ring surrounding each wound. Data are expressed as mean ± SD (*n* = 3 per group per time point); statistical comparisons were performed using one-way ANOVA with Tukey's post-hoc test (*** $p < 0.001$ vs. Model group at the corresponding time point). Data are expressed as mean ± SD (*n* = 3 mice per group per time point; different mice were used at each time point, from the MEBO comparator cohort); statistical comparisons were performed separately at each time point using one-way ANOVA with Tukey's post-hoc test (*** $p < 0.001$ vs. Model group at the corresponding time point); these comparisons are exploratory.

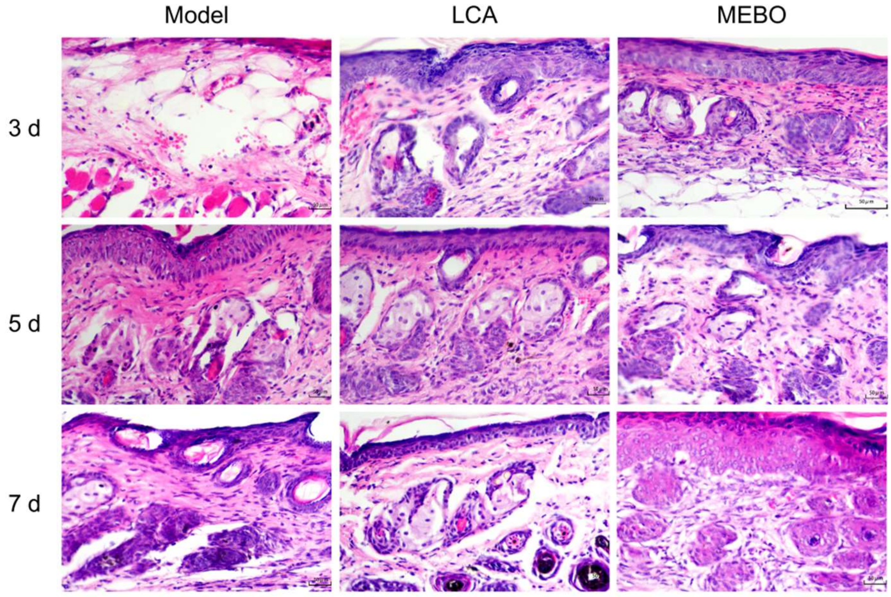

Figure S2. Histological assessment of wound skin by H&E staining in the Model, LCA, and MEBO groups at days 3, 5, and 7 post-burn injury. The Model group showed prominent edema, inflammatory cell infiltration, and delayed re-epithelialization. In contrast, wounds treated with LCA or MEBO presented earlier re-epithelialization, better-organized dermal structure, and accelerated hair follicle regeneration. Scale bars = 50 μm.
